# Architectural dissection of adhesive bacterial cell surface appendages from a ‘molecular machines’ viewpoint

**DOI:** 10.1128/jb.00290-24

**Published:** 2024-11-05

**Authors:** Olivia E.R. Smith, Tanmay A.M. Bharat

**Affiliations:** 1 Structural Studies Division, MRC Laboratory of Molecular Biology, Cambridge CB2 0QH, United Kingdom

## Abstract

The ability of bacteria to interact with and respond to their environment is crucial to their lifestyle and survival. Bacterial cells routinely need to engage with extracellular target molecules, in locations spatially separated from their cell surface. Engagement with distant targets allows bacteria to adhere to abiotic surfaces and host cells, sense harmful or friendly molecules in their vicinity, as well as establish symbiotic interactions with neighbouring cells in multicellular communities, such as biofilms. Binding to extracellular molecules also facilitates transmission of information back to the originating cell, allowing the cell to respond appropriately to external stimuli, which is critical throughout the bacterial life cycle.

This requirement of bacteria to bind to spatially separated targets is fulfilled by a myriad of specialised cell surface molecules, which often have an extended, filamentous arrangement. In this review, we compare and contrast such molecules from diverse bacteria, fulfilling a range of binding functions critical for the cell. Our comparison shows that even though these extended molecules have vastly different sequence, biochemical and functional characteristics, they share common architectural principles that underpin bacterial adhesion in a variety of contexts. In this light, we can consider different bacterial adhesins under one umbrella, specifically from the point of view of a modular molecular machine, with each part fulfilling a distinct architectural role. Such a treatise provides an opportunity to discover fundamental molecular principles governing surface sensing, bacterial adhesion and biofilm formation.

## Introduction

Bacteria are often compelled to rapidly sense and adapt to changes in their environments, which is a requirement critical to their survival in diverse settings (1–3). Environmental sensing is often performed by filamentous (long, thin and thread-like) appendages emanating from the surface of bacteria. The extended arrangement of these filamentous appendages means that they can engage with stimuli (external molecules) at locations distant from the cell surface (1–4). These appendages are usually adhesive, allowing them to directly bind to their targets, which can range from signalling molecules, abiotic surfaces, other bacterial cells in biofilms or even host cells during infection (5).

Despite the diversity of putative targets of surface appendages, there is an underlying similarity in architecture and arrangement of filamentous appendages present on bacterial cells. In this review, we consider different contexts of bacterial environmental sensing and adhesion, focusing on a few examples along the way to highlight similarities between bacterial surface filamentous appendages. Rather than discussing specific molecular mechanisms in one system, the main goal of this article is to consider surface appendages from a ‘molecular machines’ perspective, to showcase common architectural principles. For more comprehensive reviews about specific types of adhesins and surface molecules, please refer to authoritative previous works by others (6, 7). We also highlight cases where the same appendage is utilised by bacteria in multiple scenarios, showing how the underlying molecular architecture of filamentous appendages is sufficient for multiple use cases.

## Molecules mediating extracellular contact and adhesion in different contexts

### Surface sensing: binding to an abiotic surface

**Figure 1:**
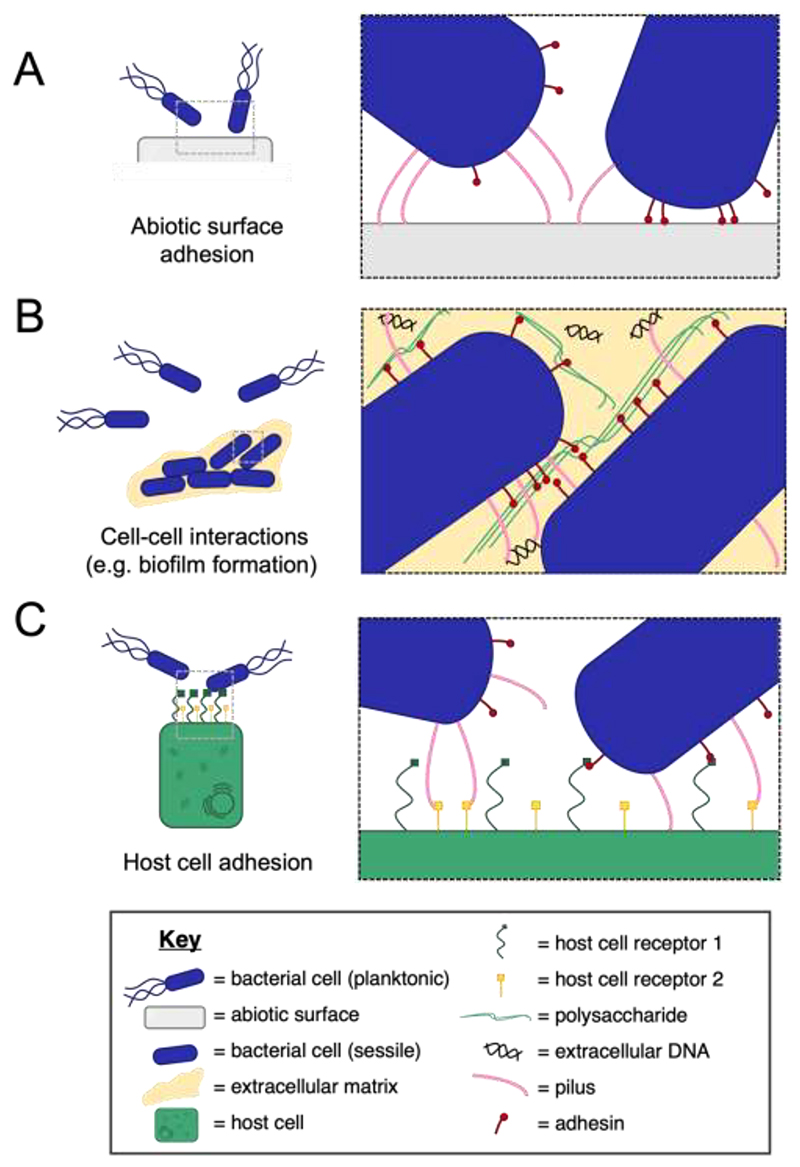
Different contexts of bacterial adhesion. Different contexts in which bacterial adhesion can occur are shown schematically (key shown at the bottom). **A)** Bacterial adhesion to an abiotic surface using multi-protein pili or single filamentous adhesin molecules e.g. *Pseduomonas aeruginosa* T4P, *Caulobacter crescentus* Tad pilus, *Paracoccus denitrificans* BapA & *Bordetella pertussis* FHA (105–108). **B)** Bacterial adhesion to ‘self’, seen specifically in the context of biofilm formation. Filamentous molecules mediating this type of interaction include the *E. coli* common pilus, *Burkholderia cenocepacia* cable pilus, *Pseudomonas putida* LapF & *Burkholderia pseudomallei* BbfA (109–112). **C)** Bacterial adhesion to ‘non-self’, seen in microbiomes or in the context of host cell infection. Examples of the filamentous molecules which interact with non-self-molecules include *Corynebacterium diphtheriae* SpaA-type pilus, *Neisseria meningitidis* T4P, *Streptococcus pyogenes* Sfb1 & *Shigella flexneri* IcsA (113–116).

Characteristic surface-attached growth as well as initial stages of biofilm formation are usually associated with the adherence of bacterial cells to an abiotic surface (8). As bacterial cell-surfaces are often charged, electrostatic repulsion results in bacteria being unable to stick easily to abiotic surfaces made of hydrophobic organic chemicals (6). Therefore, surface-located filamentous molecules specifically adapted for adhesion to a surface (Fig 1A) are required to allow bacterial cells to bind to solid surfaces (6).

A prototypical example of a class of molecules mediating surface adhesion is provided by bacterial type IV pili (T4Ps) (9). T4Ps have been characterised in a myriad of bacterial species including *Pseudomonas aeruginosa, Neisseria meninigitidis, Neisseria gonnorhoeae* and *Myxococcus xanthus*, amongst others (10). Although T4Ps fulfil diverse roles including host cell interactions, a prominent role of T4Ps is mediating bacterial adherence to abiotic surfaces during the nascent stages of biofilm formation (11). In *M. xanthus*, T4Ps facilitate bacterial movement over solid surfaces to nutrient-dense regions for subsequent colony formation (12–14). This movement across surfaces is known as twitching motility and is facilitated by the characteristic dynamic nature of T4Ps (15, 16). T4Ps are anchored to the cell surface of the bacterium via a type IV secretion platform. In *M. xanthus* this consists of proteins PilCOMNPQ and TsaP (17–20). The *M. xanthus* T4P is made up of multiple protein subunits of the pilin molecule PilA that extend from the cell surface (21). At the tip of the pilus, the PilY1 protein is located, which both primes the pilus for extension and facilitates adhesion (19). Finally, the pilus can dynamically extend and retract in an ATP-dependent manner, governed by cytoplasmic ATPases PilB and PilT. This allows the adhered bacteria to move towards the surface (17, 22).

Twitching motility is also observed in other Gram-negative bacteria including *P. aeruginosa, N. gonorrhoeae, N. meningitidis*, where it facilitates biofilm and microcolony formation (23–25). Although not discussed in detail in this article, Gram-positive bacteria similarly use T4Ps for surface adhesion. For instance, *Clostridioides difficile* uses a T4P to facilitate binding to abiotic surfaces, where it can also exhibit twitching motility (26).

While T4Ps are an example of a long-range bacterial filamentous appendage that can extend several microns away from the cell, there are other appendages that can bind to targets in closer proximity to the bacterial cell surface. For instance, LapA is a cell-surface associated filamentous adhesin which has also been shown to mediate abiotic surface binding in environmental pseudomonads including *P. fluorescens* and *P. putida* (27–29). LapA is a 519 kilodalton-sized adhesive protein, which is arranged as multi-domain polypeptide with 37 repeated domains, each approximately 100 amino acid residues in length at the N-terminal part of the protein (30). The N-terminal part of LapA is in turn anchored at the cell-surface through a type II secretion system which consists of multiple accessory proteins (31–34). There are adhesive domains in the C-terminal region of LapA that consist of a Calx-β-domain, a von Willebrand Factor Type A domain and seven repeat-in-toxin (RTX) domains which are all suggested to contribute to LapA’s function in binding to abiotic surfaces (30, 35). Atomic force microscopy has shown a two-fold decrease in adhesion of cells without LapA displayed on their surface (36). Although beyond the scope of this review, similar single polypeptide filamentous adhesins are observed in several bacteria. For example, the 600-nm long, 1.5 megadalton-sized *Marinomonas primoryensis* ice-binding protein (MpIBP) adhesin facilitates the positioning of the bacterium in an advantageous niche in the environment at the top of the water column, where ice is found, and which is rich in nutrients and oxygen (37).

Although the two examples highlighted above facilitate bacterial adhesion over different length scales – namely, long-range adhesion by T4Ps or short-range adhesion by the polypeptide LapA - both share certain architectural characteristics. The middle part of the appendage that we refer to as an extension module is noticeably present and required in both cases. This extension module allows the appendage to stretch from the cell surface, exit the polysaccharide-rich surface of the bacterium, reaching into the extracellular milieu where interaction with a target (in this case an abiotic surface) can occur. In the case of T4P, this extension module consists of multiple protein monomers of PilA, while in the case of LapA, 37 tandemly arranged N-terminal domains carry out this function of bridging the cell surface to the target molecule through the repeated domains (Fig. 1A).

### Biofilm cell-cell interactions: binding to self

After initial attachment to an abiotic surface, many bacteria form surface-attached multicellular communities called biofilms (38). As the biofilm develops, cell-cell interactions become important for holding cells together and maintaining the structural integrity of the multicellular community (Fig 1B). In mature biofilms, both long-range multi-subunit pili, as well single polypeptide filamentous adhesins play important roles in mediating these interactions (39–41).

Cell-cell interactions in *P. aeruginosa* biofilms are facilitated through numerous molecular mechanisms, however the so-called archaic Chaperone-Usher pili (CUPs) play a central role in biofilm maturation (42). In particular, CupE pili from *P. aeruginosa* contribute to the mushroom shape of biofilms and aid in colony formation, stabilising the three-dimensional architecture of the multicellular community (43, 44). CupE pili are made of multiple subunits of the CupE1 protein that each extend a so-called donor strand into the following subunit, forming an extended fibre structure (45). CupE pili are anchored in the outer membrane of the bacteria by usher protein CupE5 (45). At the tip of the CupE pili, the CupE6 adhesin has a predicted exposed hydrophobic groove, which could be important in binding to apolar substrates that are rich in the extracellular matrices of biofilms (45).

A similar principle is observed in other bacteria, for instance, *Acinetobacter baumannii* uses Csu pili to mediate cell-cell interactions in the developing biofilm (46). Deletions in the assembly apparatus of this pilus resulted in strains being unable to form mature biofilms on a range of surfaces (46). In a related mechanism of biofilm stabilisation, *Clostridioides difficile* also uses T4Ps to interact with extracellular DNA (eDNA) in biofilms, which stabilises the multicellular community (26). Slightly removed from the biochemical adhesion mechanism employed by appendages discussed in this article, we draw attention to bacterial functional amyloids (47) that stabilise biofilms in a biophysical mechanism involving filament aggregation (39, 48–52).

In addition to the long-range filaments such as CupE pili that support the assembly of mature biofilms, *P. aeruginosa* employs the important filamentous adhesin CdrA to mediate interactions between cells in biofilms. CdrA is a 220 kilodalton-sized multi-domain polypeptide (53) that protrudes 70 nm from the cell surface into the extracellular matrix (54). CdrA contains several MBG2-type repeat domains that allow it to extend from the outer membrane (55). CdrA is a two-partner secretion system adhesin that is anchored on the cell surface by its partner protein CdrB, which forms a β-barrel in the outer membrane (56). Being anchored in the CdrB pore at its C-terminus, the N-terminus of CdrA contains an adhesive tip that interacts with the mannose-rich, long-chain polysaccharide Psl (57, 58). This interaction is crucial for biofilm stability as blocking it disassembles biofilms (54). In the same vein as *P. aeruginosa* CdrA, other bacteria use other polypeptide adhesins to mediate cell-cell interactions in biofilms, such as the Ag43 protein of *Escherichia coli*, which forms zipper-like dimers to tether opposing cells together, leading to the formation of cellular rosettes (59, 60).

Beyond the spatial access provided by the appendage’s extension module highlighted in the previous section, both the long-range multi-protein pili and the short-range polypeptide adhesins share additional common design principles (Fig. 1B). In particular, both CupE pili and CdrA adhesins are anchored at the surface of the bacterial cells through a defined molecular interaction at one of the ends of the appendage, which we refer to as an anchoring module. In the case of CupE pili, the pilus is anchored to the CupE5 usher, while in the case of CdrA, the C-terminus is held in the outer membrane barrel of CdrB using a cysteine hook (56). In Gram-positive bacteria likewise, both multi-protein pili and multi-domain adhesins have a sorting signal (typically a conserved LPXTG motif) which facilitates anchoring of the appendage into the cell wall via action of sortase enzymes (61, 62). Overall, an anchoring module is present in all bacterial surface appendages, making it an important characteristic architectural feature, even though the biochemical mechanisms of anchoring differ markedly from case to case (63, 64).

### Non-self-interactions: binding in host infection

For pathogenic bacteria, the ability to colonise host tissue is often dependent on the ability to recognise and bind to host cell surface proteins (Fig 1C). This is a crucial part of the establishment of infection and the specificity of adhesion dictates tissue tropism and the colonisation of a specific niche (65).

The FimA pilus of *Porpyhromonas gingivalis* is a crucial virulence factor that facilitates both biofilm formation and host cell colonisation, causing chronic periodontal disease (66). This distinctive type V (type 5) pilus is composed of many copies of the FimA subunit, creating a 0.3-1.6 µm long filament (67). FimA subunits polymerise and extend through a protease-mediated strand exchange mechanism, where the action of Rgp proteases results in release of a β-strand to fill a hydrophobic groove of the neighbouring subunit, generating the polymeric pilus (68, 69). This mechanism is distinct from the donor-strand complementation mechanism operating in the assembly of type 1 pilus subunits that results in a different pilus morphology (68, 70). In the case of the FimA pilus of *P. gingivalis*, the proximal FimA subunits are tethered to the cell by the FimB protein (71). The adhesive tip of the protein is formed by the distal FimA subunit with the accessory proteins FimC, FimD and FimE (72, 73). The FimA pilus has been shown to bind diverse targets, including proline-rich salivary proteins, statherins and host-cell associated matrix proteins (74–79). These host-cell associated proteins include keratin, integrin, collagen type-I, fibronectin, laminin and elastin (79–83). Furthermore, the tip accessory proteins have been shown to have an important role in binding, with deletion of FimCDE attenuating binding to fibronectin and type-I collagen (84). A similar principle is observed in the Fim (type 1) pilus of uropathogenic *E. coli* where the distal FimH tip protein interacts with terminal α-D-linked mannose residues of N-linked glycans on urinary tract epithelial cells (85, 86). Other examples of adhesion include flagella in *P. aeruginosa* and *Clostridioides difficile*, which have been found to promote adherence to epithelial cells and human mucus during infection (87–90). Specifically, the flagellar cap protein (distal tip of the flagellum) has been implicated in mediating interactions with the target in both cases (89, 91).

Besides long pili such as FimA from *P. gingivalis*, single polypeptide adhesins can also support host colonisation. A notable example is provided by the fibronectin binding protein A (FnBPA), which is a crucial mediator of host colonisation in *Staphylococcus aureus* (92, 93). FnBPA is anchored to the cell surface of *S. aureus* cells via a C-terminal LPXTG motif that links to 11 extended repeated regions that position the N-terminus away from the cell surface (61, 94). The N-terminal section of FnBPA consists of three immunoglobulin-like (Ig-like) domains, which form a promiscuous ligand-interacting site that can attach to the structurally distinct eukaryotic extracellular matrix proteins fibrinogen, fibronectin and elastin (95). At the molecular level, adhesion is mediated by a “dock, lock and latch mechanism”. This involves a fibrinogen peptide inserting into an open trench between two of the Ig-like domains, catalysing a conformational rearrangement that locks the bound peptide in place (96, 97). This strong interaction can also take place in two different scenarios; either through interaction with fibronectin in the extracellular matrix or through fibronectin bound to integrin (98). In *S. aureus* cells lacking FnBPA, the ability to colonise host cells is reduced by approximately 500-fold (99, 100). In addition to host colonisation, FnBPA has also been shown to be crucial in *S. aureus* biofilm formation, suggesting multiple functions of the same protein (101, 102).

Multi-protein host binding pili like FimA and single polypeptide adhesins like FnBPA share all the molecular machine construction features mentioned above, including a cell-anchoring module at one of their ends, as well as multiple repeat domains that form the extension module, allowing the appendage to exit the immediate capsule of the bacterium. Another feature highlighted above is the presence of an adhesin at the distal end of the appendage, which we call an adhesion module. This module is positioned at the end away from the cell surface that engages with the target molecule. In FimA pili, this adhesion module consists of both the FimA subunit and the FimCDE accessory proteins while in FnBPA it is the fibronectin binding N-terminal immunoglobulins.

## Common features of a prototypical filamentous adhesive appendage

**Figure 2:**
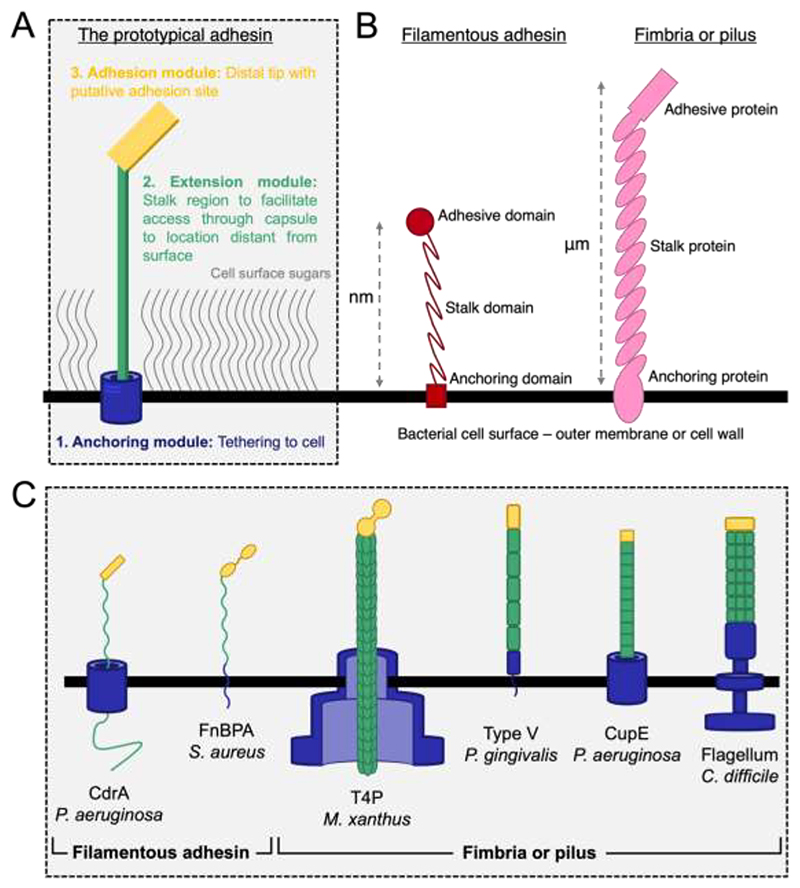
Architectural dissection of microbial filamentous surface adhesive appendages. **A)** The molecular architecture of a prototypical microbial cell surface adhesive appendage. We propose that all surface appendages must contain three modular parts; first, the presence of an anchoring module which ensures that the appendage remains bound to the cell. Second, an extension module comprised of repeated domains or polymeric proteins enables the appendage to extend away from the cell and out of the dense, sugary network at the cell surface. Third, at the distal end of the appendage, far away from the cell surface, an adhesion module mediates interactions with target molecules. **B)** The comparison between a single polypeptide filamentous adhesin and a multiprotein pilus. Despite differences in length, biochemistry and molecular details, the overall modular molecular architecture of every appendage is the same from a “molecular machines” perspective. We propose that all appendages (fibrillar polypeptides, pili or fimbriae and even polysaccharides) must contain the three basic modules highlighted in panel A. **C)** The overall molecular architecture of examples of a few filamentous appendages described in the text. The colours are the same as panel A to highlight the architectural modularity. Despite differences in molecular details, there is an underlying simplicity in the molecular architecture of filamentous surface adhesive appendages. Panel made using information from (21, 45, 68, 95, 117, 118).

In all contexts of extracellular interactions, we have found common design features that are shared by filamentous microbial appendages. We have highlighted one of these design features in each of the sections above, and our enumeration of these characteristics allows us to define common features that must be shared by every filamentous appendage (Fig. 2A). In addition to the examples discussed in detail above, we provide further examples of filamentous appendages in Table 1, all of which exhibit the same architectural principles discussed in this review.

First, every appendage must have an anchoring module: a part of the machinery dedicated to affixing the appendage at the cell surface to maintain connection with the underlying cell, as well as to facilitate the potential transfer of information from distal locations back to the bacterium. Second, all appendages contain an extension module: a part of machinery dedicated to stretching from the cell surface through a repeated stalk region. This is facilitated by structurally repeating domains in a single polypeptide adhesin or by many copies of the same protein in a multi-protein pilus or fimbria. Third, the distal tip of the appendage (located far away from the cell surface) must contain an adhesion module which facilitates binding to a surface or to a ligand. All these features are found unanimously across all the surface appendage examples that we have discussed in this review, and we propose in others that we have not considered in detail (see also Table 1). These requirements usually dictate a certain modularity that must be built into bacterial surface appendages. The molecular architecture of each appendage must therefore simultaneously fulfil the multiple requirements of anchoring (to the cell surface), extension (from the cell surface) and adhesion (to a distant target). Irrespective of underlying molecular details, be it multi-protein pili or single polypeptide filamentous adhesins, and also irrespective of biological function, i.e. binding to abiotic surfaces, to other bacteria or to hosts, these requirements of anchoring, extension and adhesion must be fulfilled, meaning that the governing molecular principles are the same in all appendages from a ‘molecular machines’ viewpoint (Fig 2B-C).

## Diversity of adhesion in bacteria

Overall, we have discussed how different types of proteinaceous bacterial surface appendages play an important role in adhesion in numerous contexts. These adhesive appendages share the same overall modular architecture, as we have asserted in this article. Several examples further demonstrate a large degree of redundancy in the functional roles of different filamentous appendages. For example, a filamentous appendage could be involved both in biofilm formation and in binding to the host in infection (Table 1 and Fig. 2). This observation of redundancy further supports the idea of underlying simplicity in the molecular architecture of surface appendages.

Both from a microbiological, as well as therapeutic perspective, understanding bacterial adhesive appendages is of particular importance. Surface appendages can have a profound influence on the fundamental cell biology of bacterial cells, because the expression of these appendages can rapidly drive planktonic bacteria into biofilms and facilitate surface colonisation (53). From a therapeutic standpoint, these appendages are often virulence factors and targets for the design of antimicrobials. For example, blocking of the *P. aeruginosa* CdrA has been shown to render biofilms susceptible to antibiotics (54). In the same manner, blocking of type 1 pili in uropathogenic *E. coli* has also been shown to decrease cell adhesion and biofilm formation (103).

We propose that these design principles could be extended to all surface appendages; for example, even non-proteinaceous appendages could be considered within the same molecular framework, possessing a modular architecture with specific design features (Fig. 2A). Let us consider the example of the dedicated molecular machinery for surface adhesion in *Caulobacter crescentus* called holdfast, which is an adhesive polysaccharide appendage. The holdfast polysaccharide is anchored just beneath the outer membrane through the HfaABD complex, while extension into the extracellular space, as well as adhesion are carried out by polysaccharide components of the holdfast (104). Even this primarily non-proteinaceous adhesive appendage can be conceptually deconstructed into the three basic architectural modules. Inevitably of course, there are variations in the precise molecular details in each system, however, it is helpful to think of bacterial adhesion from this perspective to delineate common features.

In summary, there is an underlying shared molecular architecture in all bacterial filamentous appendages, which is not confined to appendages that share the same sequence or function. This underlying modular architecture is repeated in many examples of molecules that facilitate bacterial adherence to extracellular targets, highlighting the importance of environmental sensing and adhesion in bacteria. Interacting with distant targets is an important capability of any cell, beyond even bacteria, which is required for the cell to adapt to a rapidly changing environment. The usage of filamentous appendages containing three basic modules by cell surfaces represents a general modular solution to the universal problem of organising molecular interactions outside the periphery of a cell, which we propose is pervasive across all (micro)organisms.

## Figures and Tables

**Table 1:** Further examples of filamentous surface adhesive appendages along with their architectural modules.

Name and type	Species	Anchoring Module	Extension module	Adhesion module	Role(s)	References and further reading
Fim pilus (Type 1 - chaperone-usher pilus)	*Escherichia coli*	FimD usher adds FimA units into a rod and anchors the rod in the outer membrane.	Made of up to ~3000 copies of FimA in ~2 μm long pili. Rod extends through donor-strand complementation between FimA subunits.	Adhesive protein FimH is associated with FimG and FimF at the distal tip of the pilus.	Host cell binding to uroplakin 1a receptors (mannose moieties in N-linked glycans) in the urinary tract during infection.	(70, 85, 86, 119–123)
Thin aggregative fimbria (Tafi) (Nucleation-precipitation pilus)	*Salmonella enterica*	AgfEFG proteins regulate fibre assembly and maintain the fidelity of fibres.	Fibre consists primarily of repeating units of AgfA (major fibre component) with a predicted the β- strand rich structure associated with nucleation-precipitation pili. AgfB is a minor fibre component.	Undefined adhesion module.	Associated with cellulose and other extracellular polymeric substances in biofilms.	(124–128)
Type IV pilus (Type IVa pilus)	*Pseudomonas aeruginosa*	Type IV secretion platform, consisting of the proteins PilCFMNOPQ, anchors the pilus to the cell surface. The cytoplasmic ATPases PilT, PilB and PilU govern the ATP- dependent pilus extension and retraction.	Pilus made of multiple subunits of pilin PilA. T4P dynamically extends and retracts through polymerisation and depolymerisation of PilA.	PilY1 protein mediates the adherence to abiotic surfaces at the distal tip.	Mediates interactions with surfaces in biofilm formation.	(10, 129– 137)
Fine tangled pilus (Ftp) (tad pilus)	*Haemophilus ducreyi*	Undefined anchoring module.	Comprised predominantly of pilin subunit FtpA.	Not much known about the adhesive tip of FtpA.	Adherence to abiotic surfaces. Microcolony formation: interaction with self.	(138–140)
Toxin-coregulated pilus (TCP) (Type IVb pilus)	*Vibrio cholerae*	TcpC forms a secretin ring in the outer membrane and is associated with TcpQ and TcpS. TcpDEJRT form the inner membrane-associated anchoring machinery. Incorporation of the minor pilin TcpB, rather than TcpA, at the inner membrane causes retraction of the pilus.	Formed from repeating subunits of the pilin TcpA.	TcpB (minor pilin) forms a trimer at the pilus tip, creating the adhesion module. Association of TcpB and TcpF at the distal tip alters conformation allowing the pilus to leave the outer membrane through the TcpC secretin ring and extend.	Microcolony formation: aids in colonisation of the human intestine.	(141–144)
Tad pilus (Type IVc pilus)	*Caulobacter crescentus*	Tad secretion system. Pilus retracts upon cell surface detection.	Tad pilins (PilA) form the extension module. Unlike other type IV pilins, Tad pilins lack the characteristic β- sheet rich globular domains.	Undefined adhesion module.	Abiotic surface adherence. Biofilm formation.	(145, 146)
Pilus Islet 2 (PI-2) pilus (Sortase- dependent pilus)	*Streptooccocus oralis*	Unlike other Gram-positive pili, lack a basal pilin to link the pili to the cell wall. A housekeeping A-type sortase recognises the PitB pilin for attachment to the cell wall. Molecular mechanism for anchoring and length control is not fully known.	Many copies of the PitB pilin.	PitA pilin forms the distal tip of the pilus.	Coaggregation with Acinetobacter oralis in dental plaque (biofilm) formation. Interaction with galactose.	(147–149)
Emp pilus (Sortase- dependent pilus)	*Enterococcus faecium*	Assembled at the cell surface and integrated into the cell wall using a sortase.	Composed of the subunits EmpA, EmpB and EmpC. EmpC is the major pilin.	EmpA localises to the tip of the fibre where it plays a key role in regulating the length of the pilus. All three pilins shown to be important in the interaction with extracellular matrix proteins. EmpA and EmpB indispensable in mediating biofilm interactions.	Biofilm formation. Host cell interactions in urinary tract infections: interactions with extracellular matrix proteins.	(147, 150– 153)
Hap (Monomeric autotransport er - type V secretion system)	*Haemophilus influenzae*	Hap consists of 3 major domains: an N-terminal signal peptide, a passenger domain & a C- terminal outer membrane translocator domain. The C-terminal domain is also an outer membrane β-barrel which holds Hap at the cell surface.	A section of 60 residues separates the outer membrane barrel from the passenger domain. The passenger domain is elongated, contributing to extension from the cell surface.	The passenger domain acts as the adhesion module. Within this domain, the extracellular- matrix binding region facilitates binding to matrix components. The SAAT region facilitates adherence to epithelial cells and self (Hap-Hap interactions).	Binds to matrix components fibronectin, laminin and collagen IV. Microcolony formation and biofilm formation via Hap-Hap interactions.	(154–157)
LecB	*Pseudomonas aeruginosa*	Associated with the membrane.	Homotetramer which consists of 4 LecB monomers.	Contains a calcium-dependent carbohydrate- binding domain.	LecB binds to biofilm matrix sugars to stabilise the multicellular community.	(158, 159)
FrhA	*Vibrio cholerae*	Two-partner secretion system consisting of two proteins: FrhA and FrhC. FrhC is an outer membrane pore which holds the FrhA adhesin.	Extension module is not well- characterised.	FrhA is the adhesin molecule and contains a C- terminal peptide binding domain. Also contains four cadherin repeats that help binding to epithelial cells and erythrocytes.	Host cell binding. Biofilm formation.	(160–162)
Ace	*Enterococcus faecalis*	Held at the surface of cells through the action of sortases to integrate its C- terminal LPXTG anchoring motif into the cell wall.	The B domain of Ace acts as the extension module and consists of a variable number of repeats (between 2 to 5 repeats of 47 amino acids).	Binding to collagen requires the A domain of Ace, which has two sub-domains with an Ig-like fold. Mechanism of binding is known as a ‘collagen hug’ and is an adapted version of the dock-lock-latch mechanism employed by other MSCRAMMs.	Interaction with host cell- associated molecules. Adherence of cells to human collagen type IV and dentin (and rat collagen type I and mouse laminin).	(163–166)
SdrG	*Staphylococcus epidermis*	SdrG is held at the cell wall with a proline-rich cell wall spanning domain which also contains the characteristic LPXTG motif for tethering to the cell wall.	The R domain consists of serine and aspartate repeats to extend the ligand domain away from the cell surface.	The N-terminal ‘A’ domain employs the canonical dock-lock-latch mechanism to bind to fibrinogen.	Fibrinogen binding – host cell interaction.	(96, 167– 169)
Aap	*Staphylococcus epidermis*	C-terminal LPXTG motif binds to the cell wall.	Aap is a ‘periscope protein’ with a variable number of repeats in the “B” domain.	“A” domain is a lectin-binding domain.	Binds to polystyrene. Interaction with corneocytes (host interaction). Biofilm formation.	(170–172)
BabA	*Helicobacter pylori*	The second domain is predicted to form an outer membrane-spanning β-barrel for anchoring BabA.	Undefined extension module.	Extracellular adhesin domain consists of a β- sheet region that interacts with fucosylated blood group antigens.	Host cell binding to fucosylated blood group antigens on gastric mucosa e.g. Lewis^b^ antigen.	(173–176)
YadA (trimeric autotransport er adhesin – type V secretion system)	*Yersinia pestis, Yersinia pseudotuberculo sis, Yersinia enterocolitica*	Each monomer consists of a β- barrel as the membrane-anchoring domain, forming a trimeric β-barrel together with two other subunits.	Each monomer has a coiled-coil stalk which form a trimeric coiled coil together and make the extension domain.	The ligand binding domains of each of the polypeptides have a left-handed parallel β-roll fold and together form a compact domain.	Host cell binding: interaction with collagens, fibronectin and laminins in the matrix. Autoagglutinati on.	(177–180)
Filamentous haemagluttini n (FHA) (Haemaggluti nin-like adhesin – type V secretion system)	*Bordetella pertussis*	Two-partner secretion system consisting of two proteins: FhaC and FHA. FHA N-terminus interacts with the FhaC pore to anchor at the cell surface.	FhaB is the ~370 kDa precursor to the extended β-helical FHA that is cleaved upon exit from the FhaC pore.	The adhesive domain interacts with ligands on tracheal cells including complement receptor 3 and very late antigen V.	Host cell binding.	(181–185)
